# Tight cultures as a double-edged sword for burnout: the job demands-resources perspective on perceived organizational tightness and the moderating effect of gender

**DOI:** 10.3389/fpsyg.2025.1642855

**Published:** 2026-01-07

**Authors:** Shota Kawasaki, Xi Zou

**Affiliations:** 1 Department of People and Organisations, NEOMA Business School, Reims, France; 2 Leadership, Management, and Organisation, Nanyang Business School, Nanyang Technological University, Singapore, Singapore

**Keywords:** burnout, disengagement, exhaustion, gender, organizational tightness

## Abstract

**Introduction:**

Although research on cultural tightness—the extent to which groups enforce strict norms and tolerate few deviations—reveals mixed effects on wellbeing, most existing evidence comes from national or regional contexts. Yet the dynamics inside organizations are distinct and warrant direct examination, as tight organizational cultures shape employees’ daily job demands and resources. Building on cultural tightness theory and the job demands-resources model, we examine how perceived organizational tightness impacts employee burnout by influencing job demands and resources and whether these pathways differ by employee gender.

**Methods:**

We conducted a daily diary study across one workweek with 415 participants in the United Kingdom (Study 1) and a pre-registered online experiment with 643 participants in the United States (Study 2). We tested our hypotheses using multilevel regression analyses and path analyses in Study 1 and analysis of variance in Study 2.

**Results:**

The results indicated that perceived organizational tightness was associated with higher job demands and greater job resources, which then led to increased exhaustion and reduced disengagement, respectively. Further, the link between perceived organizational tightness and job demands was stronger among women, whereas the link between perceived organizational tightness and job resources was stronger among men.

**Discussion:**

This research clarifies why tight organizational cultures can both protect and undermine employee wellbeing and offers guidance for creating conditions that reduce burnout for both women and men.

## Introduction

Cultural tightness, defined by strong norms and low tolerance for violations (Gelfand, 2018), plays a pivotal role in shaping employee behaviors to align with organizational expectations in order to achieve collective goals. By enforcing clear rules and imposing penalties for deviance, tight cultures drive behavioral convergence and uniformity among employees (Di Santo et al., 2021; Gelfand et al., 2006; Li and Gelfand, 2022). However, the cultural impacts of such practices on wellbeing present a far less uniform pattern, revealing positive, negative, and curvilinear effects. On the positive side, cultural tightness can foster order and cohesion within groups, shielding individuals from external threats and alleviating stress associated with uncertainty (Di Santo et al., 2021; Gelfand et al., 2006, 2011). Yet, the narrow range of acceptable attitudes and behaviors limits self-expression and forces individuals to constantly regulate their actions to fit group expectations, resulting in psychological strain, diminished autonomy, and reduced subjective wellbeing (Gelfand et al., 2011; Harrington and Gelfand, 2014). Additionally, some researchers have found a curvilinear relationship, suggesting that an optimal balance between tightness and looseness is necessary to improve wellbeing (Harrington et al., 2015).

These mixed findings on the relationship between cultural tightness and wellbeing raise three critical questions. Firstly, given that cultural tightness can have both positive and negative effects, what underlying mechanisms drive these contrasting outcomes? Second, could individual differences serve as moderators that explain why cultural tightness positively affects wellbeing for some employees but negatively impacts others? Lastly, existing research on cultural tightness and wellbeing has primarily been conducted at the group level, focusing on average outcomes across cultural contexts (Chua et al., 2019; Gelfand et al., 2011; Harrington and Gelfand, 2014). However, it remains unclear whether employees within the same organization experiencing similar degrees of cultural tightness may differ significantly in their wellbeing due to individual differences.

To clarify the effects of cultural tightness on wellbeing, the current research draws on the job demands-resources (JD-R) model (Demerouti et al., 2001). The JD-R model provides a comprehensive framework for understanding how job characteristics can impact employee wellbeing. This model posits that any work conditions may be classified as either job demands or job resources, and that they represent two parallel pathways to burnout, which manifests when job demands are exceedingly high and/or job resources are threatened or lost (Bakker et al., 2014, 2023; Demerouti et al., 2001). Job demands consist of the components of work that require physical and mental effort, whereas job resources represent the components of work that support goal accomplishment and reduce the burdens associated with job demands. In light of the JD-R model, prior studies have demonstrated that contextual factors in the workplace, such as organizational climate, influence job demands and job resources, given their role in shaping management practices and employees’ work designs (Dollard and Bakker, 2010; Idris et al., 2014). Building on these prior studies, we expect that cultural tightness in the organization context (i.e., organizational tightness) shapes both job demands and job resources by establishing organizational policies and practices that ensure order and coordination among employees (Gelfand et al., 2006).

Drawing on recent research on cultural tightness (Chua et al., 2024; Wormley et al., 2021), we adopt an individual-level approach by focusing on employees’ perceived organizational tightness rather than group-level tightness. Prior research has shown that individual-level perceptions of group culture do not perfectly align with the aggregated group-level perceptions (Wan et al., 2007). Employees in the same organization may develop varied perceptions of organizational tightness based on their personal experiences with workplace rules, norms, and sanctions. Such individual perceptions directly shape employees’ daily interactions, influencing their experiences of job demands and resources, and ultimately their wellbeing.

Further, we examine how perceived organizational tightness differentially affects job demands and job resources, depending on employee gender.^1^ Previous studies have demonstrated that traditional gender roles portray men as independent and women as communal (Kite et al., 2008; Wood and Eagly, 2012), and that tight cultures intensify these stereotypical views (Gelfand et al., 2006, 2011; Toh and Leonardelli, 2012; Qin et al., 2023), creating additional pressures for women to balance agentic performance expectations with communal expectations. Consequently, when women perceive their organizational culture as tight, this perception may itself become an additional job demand, increasing their vulnerability to burnout. Building on prior evidence of divergent experiences between men and women in tight cultures, we propose that tight organizational cultures reinforce traditional gender hierarchies, exacerbating gender disparities in employee wellbeing.

**FIGURE 1:**
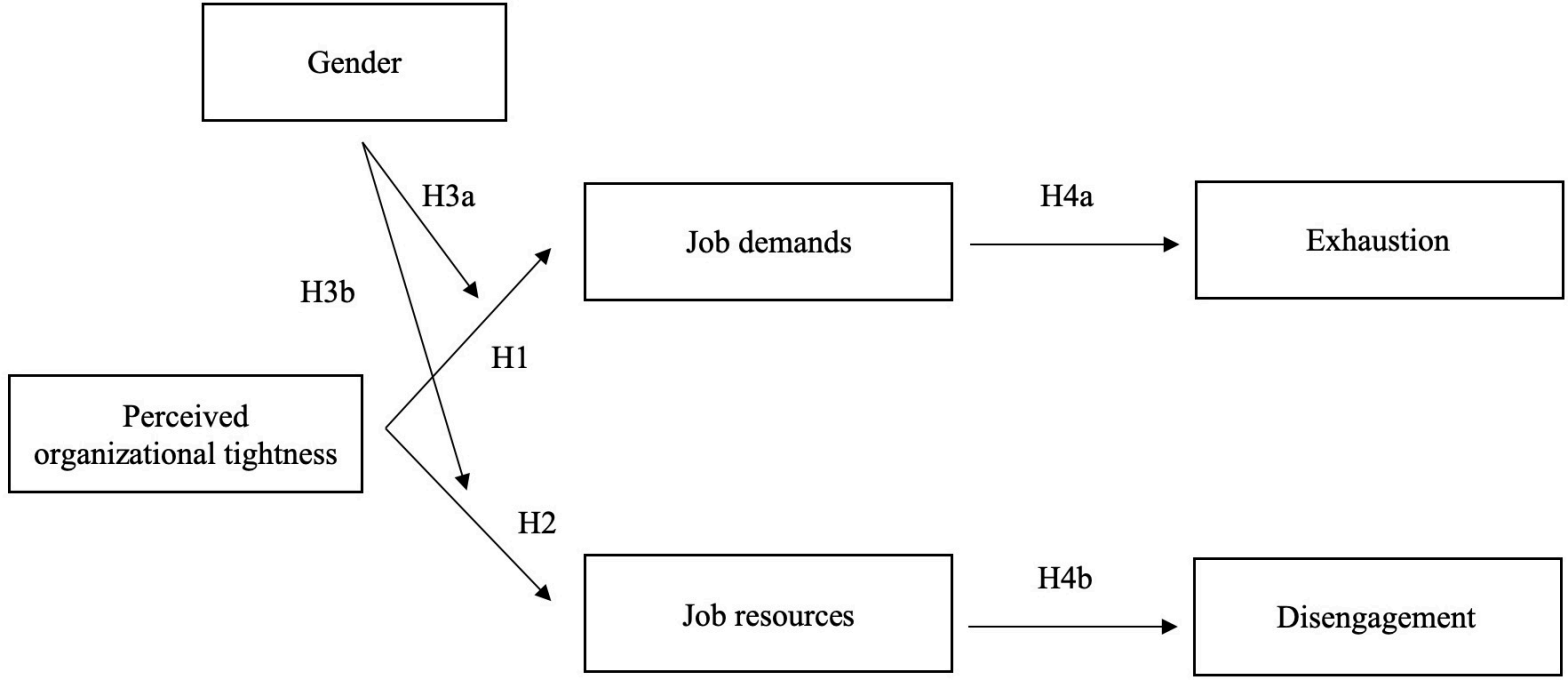
Theoretical model.

By examining how organizational tightness shapes employee wellbeing, our research contributes to the literature of cultural tightness, burnout, and gender. We identify dual pathways through which organizational tightness influences burnout: by simultaneously increasing job demands that heighten burnout and enhancing job resources that mitigate it. Further, we shed light on how organizational tightness reinforces norms that favor dominant group members, creating disproportionate demands for women to conform to male stereotypes of agency. Thus, organizational tightness increases burnout for women while creating more supportive work environments for men. By understanding how men and women experience tight organizational cultures differently through varying levels of job demands and resources, we also discuss targeted organizational cultural interventions that reduce burnout for both men and women. Figure 1 presents a full conceptual model for our research.

## The JD-R perspective to unpack the effect of organizational tightness on burnout

Research on cultural tightness highlights three perspectives regarding its impact on workplace outcomes. The first perspective emphasizes the positive effects of cultural tightness, focusing on its role in promoting social stability and cohesion. Employees in organizations with tight cultures tend to exhibit positive work attitudes such as job satisfaction and work engagement, as well as reduced stress and counterproductive behaviors (Di Santo et al., 2021; Kim and Toh, 2019; Song et al., 2023). In contrast, the second perspective highlights the negative effects of cultural tightness, particularly on creativity. Strict adherence to norms fosters fear of punishment, discouraging innovation (Harrington and Gelfand, 2014; Farmer et al., 2024; Shi et al., 2023). The presence of both positive and negative effects suggests that cultural tightness can be a double-edged sword—enhancing coordination and stability while stifling creativity and imposing psychological costs. The third perspective views cultural tightness as an amplifier that strengthens the effects of cultural norms. In tight cultures, group members internalize and enforce widely accepted norms, leading to stronger associations between their cultural values and workplace behaviors (Peterson, 2016; Yong et al., 2020). For example, Taras et al. (2010) found that Hofstede’s cultural values had a stronger predictive power for workplace outcomes in tight countries.

Exactly how does cultural tightness impact burnout? In a meta-analysis comparing national differences on burnout, Rattrie et al. (2020) reported that job demands had a marginally stronger effect on burnout in tight societies compared to loose ones. This finding aligns with the amplifier perspective, suggesting that job demands exert greater pressure on individuals in tight cultures and lead to greater burnout due to stronger normative enforcement. However, nation-level comparisons cannot determine whether the double-edged sword effect extends to individual burnout. In extremely tight cultures, employees may experience heightened stress from constantly monitoring and regulating their behavior to avoid norm violations and punishment. Conversely, in extremely loose cultures, employees may face increased uncertainty and ambiguity, which can also lead to stress.

These mixed findings about the relation between cultural tightness and wellbeing suggest that tight cultures may serve as a double-edged sword to employees, which render it particularly apt and relevant for drawing on the JD-R model to answer our research questions. The JD-R model proposes that any of the work characteristics can instigate a health-impairment (depleting one’s energy and leading to job strain and exhaustion) or motivational (satisfying work-related needs and enhancing work engagement) process to one’s wellbeing (Demerouti et al., 2001; Demerouti and Bakker, 2023). Nevertheless, the JD-R model does not precisely explain whether a specific work characteristic represents a demand or resource to employees (Bakker and Demerouti, 2017). For example, whereas strict workplace rules and policies (representing a tight culture) may be interpreted as demands that require employees to exert more mental and cognitive efforts to avoid deviance, they can be also seen as resources that provide clear guidance and reduce ambiguity around appropriate behaviors and required procedures at work. Stated otherwise, a specific work characteristic is not limited to solely representing either job demands or job resources; rather, it can serve as both.

Drawing on the JD-R model and cultural tightness theory, we argue that employees may report more job demands and resources under tighter organizational culture. In the current paper, we conceptualize organizational tightness as individual perceptions rather than a group-level construct, and do so for two reasons. First, employees experience only parts of the broader organizational culture in their everyday work. Although the original definition of cultural tightness describes societies with an overarching set of expectations, strict norms, and strong sanctions (Gelfand et al., 2006), employees may encounter these norms unevenly through their tasks, interactions, and role requirements within their organizations. Individuals therefore form subjective assessments of how restrictive norms are and how severe sanctions for norm violation might be (Wormley et al., 2021). These subjective assessments can diverge from an objective group-level culture (Wan et al., 2007), making individual-level measurement necessary. This approach is consistent with recent work that treats tightness as a perceptual construct in organizations (Chua et al., 2024; Marcus et al., 2022; Yao et al., 2025). It also aligns with research that uses situational constraints as an individual level indicator of perceived appropriateness across everyday contexts, which has been used as an alternative operationalization of tightness or looseness (Price and Bouffard, 1974; Gelfand et al., 2006; Gelfand et al., 2011). Second, the JD-R model emphasizes individual appraisal of the work environment. Employees’ interpretations of their immediate experience may be a proximal driver for their motivational and strain processes. Individual perceptions of organizational tightness are therefore more theoretically appropriate than group-level cultural tightness as predictors of the JD-R processes we examine.

### Perceived organizational tightness as a predictor of job demands

Perceived organizational tightness is likely to be positively associated with job demands because the norms, rules, and policies of tight organizational cultures act as situational constraints, adding an extra layer of job demands to employees’ experiences (Gelfand et al., 2006; Gelfand, 2018). Employees who perceive their organizational culture as tight may feel compelled to adhere strictly to protocols and formalities. For instance, strict policies about attendance and sick leave create an obligation to be present at work even when unwell, increasing job demands (Miraglia and Johns, 2016). Similarly, employees often view monitoring practices, such as desktop monitoring, video surveillance, and location tracking (Ravid et al., 2020; Thiel et al., 2023), as constraints on their autonomy, driven by the fear of punishment. Such perceptions heighten job demands by requiring constant vigilance and compliance.

Organizational sanctioning, a core feature of tight organizational cultures, further intensifies job demands. Employees often find sanctioning stressful, as it can result in negative evaluations of their competence and performance (Ng et al., 2021). To avoid such undesirable outcomes, employees must invest additional effort into monitoring and controlling their work attitudes and behaviors to conform to organizational norms. Supporting this view, research has shown that cultural tightness is associated with greater behavioral inhibition and self-control over actions and emotional expression (Church et al., 2012; Gelfand et al., 2011; Liu et al., 2018; Uz, 2015; van Hemert et al., 2007). In contrast, when employees perceive their organizational culture as loose, they become less inclined to adhere to organizational norms due to the lack of sanctioning, leading to lower job demands. Taken together, employees who perceive their organizational cultures as tight are more likely to experience higher job demands than those in loose organizational cultures.

*H1:* Perceived organizational tightness is positively associated with job demands.

### Perceived organizational tightness as a predictor of job resources

In tight organizational cultures, clear standards and information about appropriate job-related attitudes and behaviors may reduce employees’ uncertainty about work tasks, responsibilities, procedures, and priorities (Gelfand et al., 2004; Schmidt et al., 2014). As such, tight organizational cultures enhance employees’ understanding of their work roles and responsibilities by explicitly establishing organizational expectations and reducing role ambiguity. Role clarity is an essential job resource, as it prevents miscommunication, improves coordination, and promotes positive work outcomes (e.g., work engagement, organizational citizenship behavior, lower intention to leave) (Crawford et al., 2010; Eatough et al., 2011; Lyons, 1971). Importantly, the benefits of organizational tightness for job resources operate through employees’ perceptions: When employees perceive their organization as having a tight culture with clear normative expectations about job roles, they are more likely to experience a higher level of job resources. In contrast, perceiving the organizational culture as loose may increase ambiguity and reduce job resources, contributing to stress and anxiety due to unclear behavioral norms (Gelfand et al., 2006; Schmidt et al., 2014).

Further, tight organizational cultures may facilitate more information sharing and social support among employees. Prior studies have shown that compared to those in loose cultures, people in tight cultures are more likely to develop shared norms and interdependent relationships (Gelfand et al., 2011; Harrington and Gelfand, 2014), fostering social networks that enhance collaboration and information exchange. These networks create job resources by enabling employees to access advice, support, and information more effectively (Dahlander and McFarland, 2013; Schulte et al., 2012; Venkatesh et al., 2017). Employees who perceive their organizational culture as tight may be more likely to rely on these networks, reinforcing their sense of available support. Conversely, in loose organizational cultures, greater behavioral flexibility may lead to more fragmented networks, making it harder for employees to turn to their colleagues for advice and social support (Gelfand et al., 2006).

*H2:* Perceived organizational tightness is positively associated with job resources.

### The moderating role of gender on the effects of perceived organizational tightness on job demands and resources

#### Tight organizational cultures and normative expectations for men and women

As mentioned above, the JD-R model does not strictly specify whether a certain work characteristic represents a job demand or resource. This raises the possibility that the effects of organizational tightness on burnout could hinge on individual differences. Prior research has consistently documented a gender gap in burnout across occupations and countries, and the studies show that women are often more susceptible to burnout in the workplace than men (e.g., Artz et al., 2022; Bez and Emhan, 2011; Mete et al., 2022; Qamar et al., 2024; Redondo-Flórez et al., 2020). A meta-analysis by Purvanova and Muros (2010) further supports this pattern, showing that women experience higher levels of emotional exhaustion than men, regardless of whether they work in male- or female-dominant occupations. This disparity is often attributed to women facing more gender discrimination and unfair treatment in the workplace (e.g., Parker and Funk, 2017; Smith et al., 2018). Such biases not only restrict women’s opportunities for promotions and salary increases compared to equally performing male counterparts, but also impose additional stressors. As a result, women may experience a heavier emotional and psychological burden in the workplace, leading to higher rates of burnout relative to men.

Because tight cultures create strong situations and enforce uniform normative expectations for everyone (Gelfand et al., 2011; Smith, 2017), one may argue that tight organizational cultures reduce gender differences in workplace experiences. In tight organizational cultures, both men and women are held to strict behavioral expectations, potentially leveling the playing field and mitigating the factors that often contribute to higher burnout among women. However, despite such uniform normative expectations, we propose that perceived organizational tightness could, in fact, amplify the gender gap in burnout. According to social dominance theory (Ho et al., 2012; Sidanius and Pratto, 2004; Vargas-Salfate et al., 2018), normative expectations within a group tend to reflect the values and traits of dominant group members—typically men in workplace settings (Cuddy et al., 2015). This dominance is often reinforced through cultural expectations that prioritize traits that have been stereotypically associated with men—such as agency, competitiveness, and ambition—over those associated with women, such as concern for others, warmth, and relationality (Kite et al., 2008; Wood and Eagly, 2012).

As normative expectations in the workplace are often biased toward values and traits typically associated with men, we propose that tight organizational cultures may exacerbate pre-existing gender hierarchies by aligning organizational expectations with male stereotypes. According to the JD-R model, employees’ personal resources, or perceived ability to manage and control their environment (Bakker et al., 2023), buffer the negative impacts of job demands and amplify the positive impacts of job resources (Bakker and Sanz-Vergel, 2013; Xanthopoulou et al., 2009). Although organizational tightness accommodates work characteristics that could represent both job demands and job resources, women may experience greater constraints in deriving personal resources from tight organizational cultures where they must navigate normative expectations that conflict with long-held female gender roles and orientations. Such unequal expectations for men and women in tight organizational cultures may impose greater demands on women and increase their vulnerability to burnout while making it easier for men to access job resources and avoid burnout.

#### Women experience more job demands than men in tight organizational cultures

Traditionally, women have been associated with communal roles (e.g., homemakers, caregivers) that emphasize traits such as kindness and warmth (Eagly and Wood, 2016; Kacmar et al., 2011). However, as mentioned above, tight cultures often promote traits of the dominant group members—agentic traits aligned more closely with male stereotypes like competitiveness and ambition. This mismatch places additional demands on women in the workplace, as they are expected to demonstrate warmth and kindness while also displaying competitiveness and ambition. Further, when women adopt agentic traits, they risk facing gender backlash for violating traditional gender norms. Supporting this, research shows that women face harsher punishments for violating norms; for instance, while men might be rewarded for displaying anger or aggression, women are penalized for the same behaviors (Brescoll and Uhlmann, 2008; Brown and Sumner, 2006). Because tight organizational cultures may reinforce traits already associated with men, men are less likely to perceive these environments as an added burden. Consequently, perceived organizational tightness is expected to have a stronger association with job demands for women than for men.

*H3a:* The positive relation between perceived organizational tightness and job demands is stronger for women than for men.

#### Men experience more job resources than women in tight organizational cultures

We expect the positive relationship between perceived organizational tightness and job resources to be stronger for men than for women, amplifying gender disparities in accessing job resources. Indeed, job resources such as growth opportunities, leadership training, and skill development are often designed to reinforce male-dominated leadership pipelines. Research highlights that men in tight countries are more likely to emerge as leaders and to be perceived as leader-like by organizational members (Toh and Leonardelli, 2012). In the U.S., for example, states with tighter norms exhibit greater gender inequality in business and political leadership, with men disproportionately occupying top roles (Qin et al., 2023). Moreover, men with perceptions of tight cultures tend to hold stronger fairness perceptions about the world, which boosts their subjective wellbeing (Oh, 2022). Collectively, prior research suggests that organizational tightness may allow men to interpret the competitive environment as meritocratic, further legitimizing their access to job resources.

In contrast, women in tight organizational cultures may face additional barriers. Even when job resources like social support are available, these resources often carry negative social implications for women. Seeking help can be perceived as a sign of weakness (Nadler and Chernyak-Hai, 2014), leading women to internalize doubts about their own competence (Lee et al., 2023). Thus, when women perceive organizational cultures to be tight, they are less willing to ask for support, undermining their ability to leverage resources effectively (Beehr et al., 2010; Lennard and Van Dyne, 2018; Marigold et al., 2014). Such dynamics strengthen the positive relation between perceived organizational tightness and job resources for men but weaken it for women.

*H3b:* The positive relation between perceived organizational tightness and job resources is stronger for men than for women.

### The effects of job demands and resources on burnout

Integrating the propositions of the JD-R model with the hypotheses above, we propose that perceived organizational tightness impacts burnout through job demands and job resources, with the strength of the indirect effects varying by employee gender. Specifically, women who perceive tighter organizational cultures are more likely to experience higher exhaustion via increased job demands and greater disengagement via reduced job resources. In contrast, men who perceive tighter organizational cultures are likely to experience lower exhaustion and disengagement due to reduced job demands and increased job resources, respectively.

*H4a:* Job demands mediate the interactive effect between perceived organizational tightness and gender on exhaustion, such that the positive indirect effect is stronger for women than for men.

*H4b*: Job resources mediate the interactive effect between perceived organizational tightness and gender on disengagement, such that the negative indirect effect is stronger for men than for women.

## Study overview

To test our hypotheses, we conducted two studies. In Study 1, we tested the full model using a daily diary survey design to temporally separate the measures for the independent variables, mediators, and dependent variables and to address common method variance. In Study 2, we used an experimental design to strengthen the causal inference for the interaction effect between perceived organizational tightness and gender on job demands and job resources. Both studies were approved by the Research Ethics Committee at one of the authors’ institution (IRB-2020-04-017; “Daily Experiences at Work” for Study 1 and IRB-2023-349; “Organizational culture and employee wellbeing” for Study 2). Supplementary material (including items for each scale, experimental stimuli, and additional analyses), datasets, codebooks, syntax for analyses, results, and pre-registration are available on the Open Science Framework (OSF): https://osf.io/8wyrc/?view_only=76f80c4401954531a793f73f2e4416fd.

## Study 1

In Study 1, we chose a daily diary design for two primary reasons. First, we tried to reduce common method variance by collecting data for the focal variables at multiple time points with temporal separation. This is particularly important as the variables in our model were all concerned with individual perception and had to be collected through self-report. Second, as opposed to a typical cross-sectional design where individuals might base their responses on their work experiences on a specific day, the daily diary design allowed us to obtain the information about participants’ job demands, job resources, and burnout in close to real time (Bolger et al., 2003), during or right after their work had ended. We collected data over a period of one workweek so that employees’ responses would be less susceptible to possible fluctuations in their day-to-day experiences (e.g., one might experience more demands earlier in the week compared to on Friday). This study was not pre-registered.

### Participants and procedure

We recruited 495 full-time and/or part-time employees in the United Kingdom from Prolific. Participants first completed an initial survey that contained the informed consent and the questionnaires for perceived organizational tightness and demographic information. Over the following week, participants received three daily surveys. We sent the morning survey at 9 a.m., which included the measures of positive and negative affect. We sent the afternoon survey at 12 p.m., which included the measures of job demands and resources. Finally, we sent the evening survey at 5 p.m., which included the measures of exhaustion and disengagement. Participants were compensated with £2 for the initial survey and £5 per day for completing the three daily surveys.

We excluded participants who failed to pass our attention check^2^ (*N* = 17), participants with the same IP address (*N* = 2), and participants who were students, unemployed, self-employed, holding two different part-time jobs, or on furlough (*N* = 6). We also excluded daily observations where participants reported zero working hours that day (day-level *N* = 32). Retaining the daily matched observations where participants responded to all three surveys that day, we obtained a total of 1,554 day-level responses (out of 2,075 possible responses) from 415 participants (*M_*age*_* = 35.83; *SD_*age*_* = 10.00; 61.69% women; 82.41% White) with a response rate of 74.89%. Across the workweek, there were 301 matched responses on Monday, 131 on Tuesday^3^, 379 on Wednesday, 368 on Thursday, and 375 on Friday.

Our sample included 97.35% full-time employees^4^. Participants had an average of 6.07 years of experience in their current organizations (*SD* = 6.15). Participants were from various industries, such as education and training (14.70%), healthcare (10.60%), financial services (7.95%), services (6.75%), retail (6.51%), and media and entertainment (4.82%).

### Measures

The scales were presented to each participant in a separate random order, and we also randomized the items within each scale.

#### Perceived organizational tightness

In the initial survey, we measured perceived organizational tightness using the six-item measure developed by Gelfand et al. (2011). As per the prior organizational research (e.g., Di Santo et al., 2021; Kim and Toh, 2019; Song et al., 2023), the Gelfand et al.’s scale, which was originally constructed to reflect societal-level tightness, has been adapted to measure perceived cultural tightness in organizations. Specifically, we replaced “country” in the original measure with “organization” to measure organizational tightness perceived by employees. Sample items included: “There are many strong norms that people are supposed to abide by in my organization” and “In most situations, norms in my organization give people a great deal of freedom in deciding how they want to behave” (reverse-coded; α = 0.65). The items were measured on a six-point Likert-type scale (1 = *Strongly disagree*, 6 = *Strongly agree*).

#### Gender

Participants reported their gender in the initial survey (*N*_men_ = 156; *N*_women_ = 256; *N*_other_ = 3). We coded women as 0.5 and men and others as −0.5.

#### Job demands and resources

The item for job demands was “Since the last survey, I feel it is physically taxing for me to get used to my working time,” which was adapted from Schönfelder (1992) and Demerouti et al. (2001). The item for job resources was “Since the last survey, I feel I got enough feedback about the quality of my performance,” which was adapted from Hackman and Oldham (1975). We chose physical workload and feedback because they had the highest factor loading with job demands and resources, respectively, in Demerouti et al. (2001). Participants rated both items on a five-point scale (1 = *Not at all*, 5 = *Very much*).

#### Exhaustion and disengagement

We measured exhaustion and disengagement using four items adapted from Demerouti et al. (2010). We used the following two items for exhaustion: “Right now, I feel emotionally drained” and “Right now, I feel worn out and weary.” We used the following two items for disengagement: “Right now, I think less at work and do my job almost mechanically” and “Right now, I feel sickened by my work tasks.” Participants rated all items on a five-point scale (1 = *Not at all*, 5 = *Very much*). Mean inter-item correlations were 0.76 and 0.34 for exhaustion and disengagement across 5 days, respectively.

#### Control variables

Following the common practice in daily diary studies, we controlled for the effects of days (coded as an interval variable ranging from 1 to 5; Lanaj et al., 2016; Schabram and Heng, 2022). To account for individual differences, we included employees’ age, organizational tenure, and race (0 = *white*, 1 = *non-white*) as control variables, given that minorities tend to disproportionately face the risk of mental health (e.g., Miller, 2021; Stowe et al., 2022). We also controlled for the type of industries in which employees were working, as burnout is often common among certain professions that are associated with higher job demands (e.g., service workers and nurses; Nayeri et al., 2009; Taris et al., 2005). Specifically, we included dummy-coded variables for two of the industries that appeared most frequently in our samples (healthcare, and education and training). To account for the effects of daily mood on burnout, we measured morning positive and negative affect, given their associations with exhaustion and disengagement (Zellars et al., 2004). Participants rated their positive and negative moods in the morning surveys using the shortened version of the Positive and Negative Affect Schedule (Mackinnon et al., 1999). Using the stem “Right now, I feel…,” we measured positive affect with the following items: inspired, alert, excited, enthusiastic, and determined. We used the following items for negative affect: afraid, upset, nervous, scared, and distressed. The items were measured on a five-point scale (1 = *Not at all*, 5 = *Very much*; average α across 5 days was 0.90 for both positive and negative affect).

### Results

We used R version 4.4.2 (R Core Team, 2024) for our analyses. Given the nested nature of our data (daily observations nested within participants), we performed multilevel analyses using the lme4 (version 1.1-35.5; Bates et al., 2015) and lmerTest packages (version 3.1-3; Kuznetsova et al., 2017) to test our hypotheses. We grand-mean-centered perceived organizational tightness. To facilitate the comparison between men and women in our hypothesis testing, we excluded the participants who identified themselves as neither gender (person-level *N* = 3; day-level *N* = 11) and used the following effect-coding: *men* = −0.5, *women* = 0.5. We centered job demands and job resources and positive and negative affect around their respective person-level means. We also included their person-level means as level-2 predictors.

Modeling each of job demands and resources as an outcome, we first ran two models with random intercepts: First, we examined the main effects of perceived organizational tightness and gender, and then we added the interaction term between these two variables. Next, we examined the effect of job demands and resources on exhaustion and disengagement, respectively, controlling for the predictors and control variables. We performed these models with random slopes for job demands and resources in addition to random intercepts (Aguinis et al., 2013). Finally, we obtained indirect effects on the between-person level and their confidence intervals calculated by the quasi-Bayesian Monte Carlo method based on normal approximation with 10,000 resamples using the mediation package (version 4.5.0) (Tingley et al., 2014).

#### Descriptive statistics

Table 1 presents descriptive statistics and correlations for our focal variables. The proportions of total variances that reside in individuals, between-person variances or ICC(1), were not marginal for all variables: 67.63% for job demands, 73.34% for job resources, 49.46% for exhaustion, and 72.97% for disengagement. The data collected through daily surveys shows a significant amount of clustering within individual participants, confirming the need to account for the multilevel structure of the data in the analysis.

**TABLE 1:** Means, standard deviations, and correlations in Study 1.

	Variables	*M*	*SD*	1	2	3	4	5	6	7	8	9	10	11	12	13
1	Job demands	2.04	1.06	–	−0.02	0.13***	0.09***	−0.17***	0.08**	−0.07**	–	–	–	–	–	–
2	Job resources	3.07	1.26	−0.12*	–	−0.03	−0.01	0.04	−0.03	−0.04	–	–	–	–	–	–
3	Exhaustion	2.77	1.14	0.48***	−0.12*	–	0.29***	−0.09***	0.13***	−0.09***	–	–	–	–	–	–
4	Disengagement	2.14	0.90	0.51***	−0.19***	0.39***	–	−0.07**	0.13***	−0.06*	–	–	–	–	–	–
5	Positive affect	2.99	0.95	−0.23***	0.29***	−0.22***	−0.30***	–	−0.25***	0.12***	–	–	–	–	–	–
6	Negative affect	1.43	0.69	0.40***	−0.18***	0.47***	0.29***	−0.17***	–	−0.17***	–	–	–	–	–	–
7	Day	3.25	1.41	0.03	−0.02	0.00	0.10	0.08	−0.02	–	–	–	–	–	–	–
8	Perceived organizational tightness	4.36	0.60	0.11*	0.13**	0.06	0.01	0.11*	0.03	−0.02	–	–	–	–	–	–
9	Gender	0.12	0.49	0.11*	0.05	0.17***	0.02	−0.06	0.15**	−0.01	−0.01	–	–	–	–	–
10	Age	35.83	10.00	−0.07	0.00	−0.05	−0.07	0.18***	−0.03	0.09	0.17***	−0.12*	–	–	–	–
11	Race	0.18	0.38	0.03	0.07	0.04	0.08	0.01	0.07	−0.09	0.05	0.00	−0.08	–	–	–
12	Organizational tenure	6.07	6.15	−0.09	0.01	−0.10*	−0.05	0.16**	−0.02	0.05	0.09	−0.09	0.54***	−0.07	–	–
13	Healthcare industry	0.11	0.31	0.14**	−0.01	0.12*	0.06	−0.01	0.03	0.00	0.15**	0.07	0.05	−0.07	−0.10*	–
14	Education and training industry	0.15	0.35	0.11*	−0.04	0.10*	0.02	−0.09	0.14**	0.07	0.05	0.06	−0.01	0.01	−0.01	−0.14**

Level-1 *N* is 1,554 and level-2 *N* is 415. Variables 1 through 7 are within-person (Level 1) variables. Variables 8 through 14 are between-person (Level 2) variables. Within-person level correlations are presented above the diagonal. Between-person level correlations are presented below. Means and standard deviations are based on the between-person values. Day is coded as follows: 1 (Monday) to 5 (Friday). Gender is coded as follows: Women = 0.5, Men and Other = −0.5. Race is coded as follows: White = 0, Non-white = 1.

*p < 0.05.

**p < 0.01.

***p < 0.001.

#### Confirmatory factor analysis

We conducted multilevel confirmatory factor analyses to establish the discriminant validity of the variables in our model using the lavaan package (version 0.6-18) (Rosseel, 2012). We modeled our within-person-level variables (job demands and job resources, exhaustion and disengagement, and positive and negative affect) at the level 1 and level 2 and the between-person-level variable (perceived organizational tightness) at the level 2. The hypothesized model fitted the data adequately (χ^2^ = 950.64, *df* = 281, CFI = 0.94, TLI = 0.93, RMSEA = 0.04, SRMR_within_ = 0.04, SRMR_between_ = 0.08). The model fit did not improve when the items for exhaustion and disengagement loaded on a single factor representing burnout at both level 1 and level 2 (χ^2^ = 1096.02, *df* = 292, CFI = 0.93, TLI = 0.91, RMSEA = 0.04, SRMR_within_ = 0.05, SRMR_between_ = 0.09). Also, the model where all items loaded on a single factor at both level 1 and level 2 performed worse (χ^2^ = 4708.46, *df* = 313, CFI = 0.61, TLI = 0.56, RMSEA = 0.10, SRMR_within_ = 0.15, SRMR_between_ = 0.26), providing further support to the hypothesized model.

#### Hypothesis testing

**FIGURE 2:**
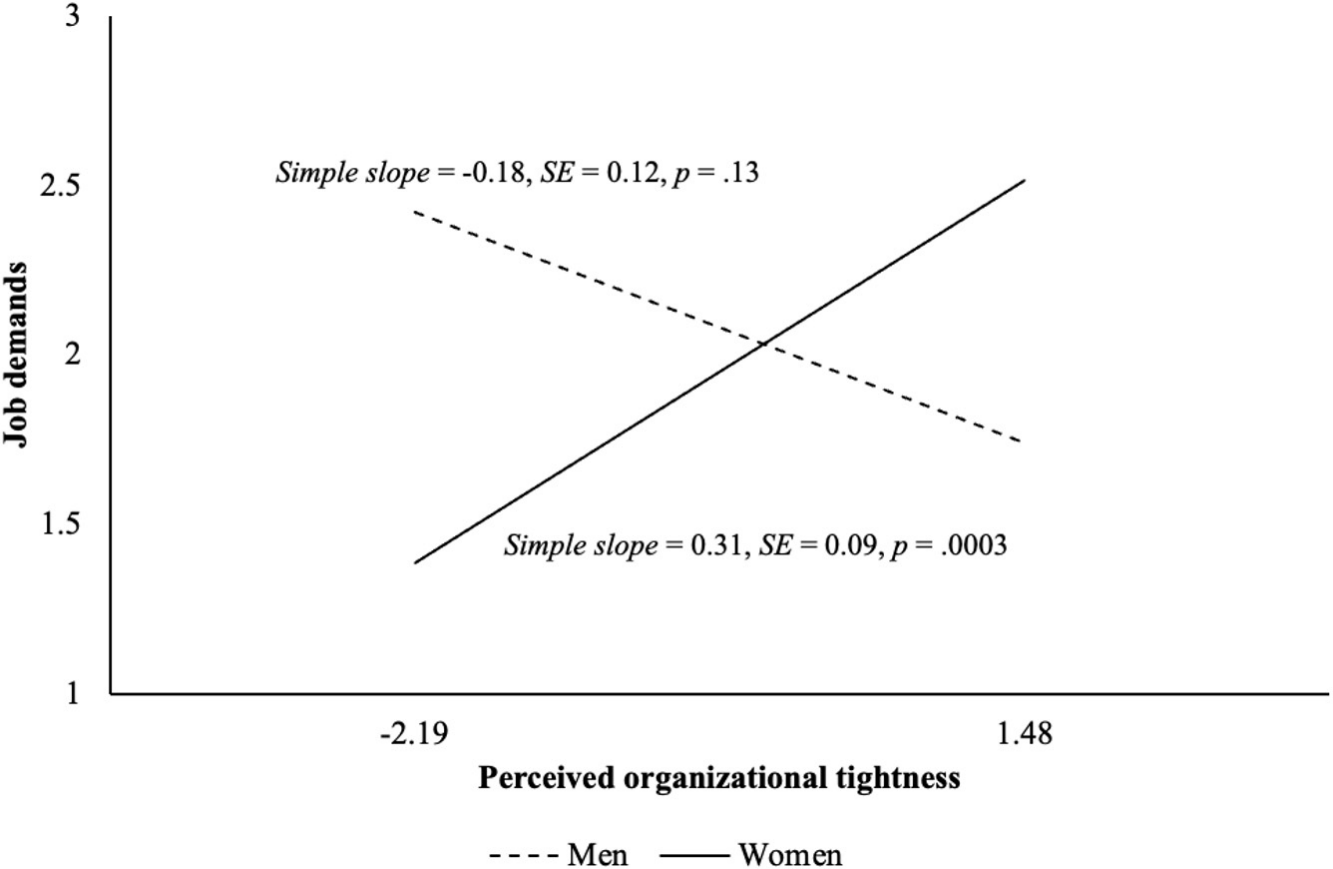
Simple slopes between perceived organizational tightness and job demands for men and women in Study 1. The slopes are depicted for the full range of grand-mean-centered perceived organizational tightness.

**FIGURE 3:**
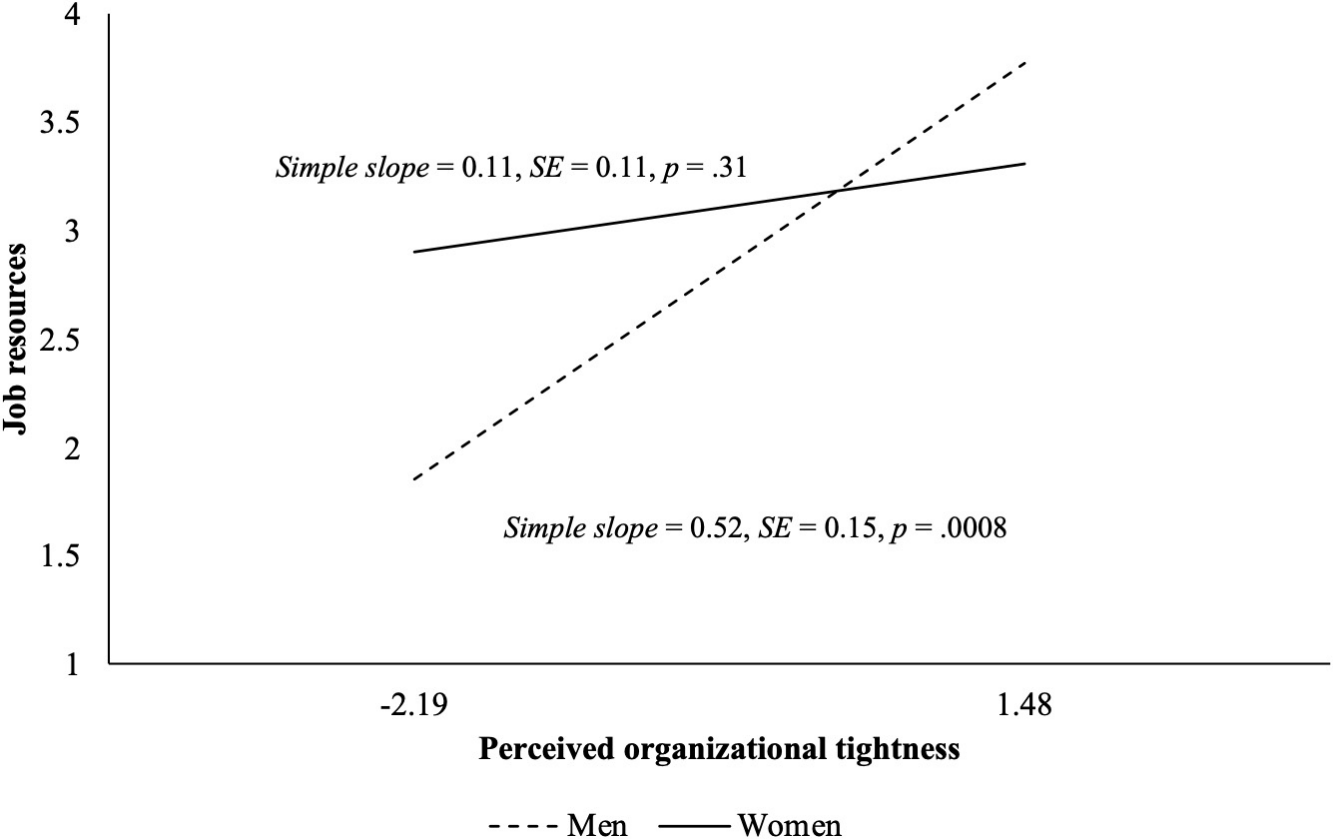
Simple slopes between perceived organizational tightness and job resources for men and women in Study 1. The slopes are depicted for the full range of grand-mean-centered perceived organizational tightness.

Table 2 presents the results of the hypothesis testing. Supporting H1 and H2, perceived organizational tightness was positively related to job demands (γ = 0.14, *SE* = 0.07, *p* = 0.043) and job resources (γ = 0.25, *SE* = 0.09, *p* = 0.006). Supporting H3a, we found that gender moderated the relation between perceived organizational tightness and job demands (γ = 0.49, *SE* = 0.15, *p* = 0.0009). The relation was positive and significant for women (*simple slope* = 0.31, *SE* = 0.09, *p* = 0.0003), but it was not significant for men (*simple slope* = −0.18, *SE* = 0.12, *p* = 0.13) (Figure 2). We also found a significant moderating effect of gender for job resources (γ = −0.41, *SE* = 0.19, *p* = 0.030). Supporting H3b, the relation between perceived organizational tightness and job resources was positive and significant for men (*simple slope* = 0.52, *SE* = 0.15, *p* = 0.0008), but it was not significant for women (*simple slope* = 0.11, *SE* = 0.11, *p* = 0.31) (Figure 3).

**TABLE 2:** Results of multilevel analyses in Study 1.

Variables		Job demands		Job resources		Job demands		Job resources		Exhaustion		Disengagement	
	γ	*SE*	γ	*SE*	γ	*SE*	γ	*SE*	γ	*SE*	γ	*SE*
Intercept	1.92***	0.26	2.73***	0.34	1.92***	0.26	2.73***	0.33	1.84***	0.25	2.83***	0.25
**Within-person level variables**												
Job demands	–	–	–	–	–	–	–	–	0.17***	0.04	–	–
Job resources	–	–	–	–	–	–	–	–	–	–	−0.01	0.02
Day	−0.02	0.01	−0.02	0.01	−0.02	0.01	−0.02	0.01	−0.03*	0.01	−0.01	0.01
Positive affect	−0.12***	0.03	0.04	0.03	−0.12***	0.03	0.04	0.03	−0.05	0.04	−0.03	0.02
Negative affect	0.04	0.04	−0.05	0.04	0.04	0.04	−0.05	0.04	0.19***	0.05	0.12***	0.03
**Between-person level variables**												
Job demands	–	–	–	–	–	–	–	–	0.29***	0.04	–	–
Job resources	–	–	–	–	–	–	–	–	–	–	−0.08*	0.03
Perceived organizational tightness	0.14*	0.07	0.25**	0.09	0.06	0.07	0.32***	0.10	0.02	0.06	0.04	0.06
Gender	0.04	0.09	0.15	0.11	0.05	0.09	0.14	0.11	0.13	0.08	−0.07	0.08
Perceived organizational tightness x Gender	–	–	–	–	0.49***	0.15	−0.41*	0.19	–	–	–	–
Age	−0.00	0.00	−0.01	0.01	−0.00	0.00	−0.01	0.01	0.00	0.00	−0.00	0.00
Race	−0.02	0.11	0.15	0.14	−0.04	0.11	0.18	0.14	0.02	0.10	0.12	0.10
Tenure	−0.01	0.01	0.00	0.01	−0.01	0.01	0.00	0.01	−0.01	0.01	0.00	0.01
Healthcare industry	0.34*	0.14	−0.03	0.18	0.27	0.14	0.03	0.18	0.15	0.12	0.11	0.12
Education and training industry	0.16	0.12	−0.05	0.15	0.12	0.12	−0.02	0.15	0.06	0.10	−0.00	0.11
Positive affect	−0.20***	0.06	0.36***	0.07	−0.19***	0.06	0.35***	0.07	−0.13*	0.05	−0.24***	0.05
Negative affect	0.57***	0.07	−0.30**	0.09	0.57***	0.07	−0.30**	0.09	0.51***	0.07	0.29***	0.07
**Variance components**												
Within-person variance	0.35	–	0.43	–	0.35	–	0.43	–	0.63	–	0.21	–
Intercept variance	0.59	–	1.00	–	0.57	–	0.99	–	0.36	–	0.48	–
Random slope variance	–	–	–	–	–	–	–	–	0.01	–	0.01	–
**Model fit**												
AIC	3588.7	–	4017.8	–	3579.6	–	4015.1	–	4169.3	–	2958.8	–
BIC	3668.8	–	4097.9	–	3665.0	–	4100.5	–	4270.8	–	3060.3	–
Log-likelihood	−1779.3	–	−1993.9	–	−1773.8	–	−1991.5	–	−2065.7	–	−1460.4	–

Level-1 *N* is 1,543 and level-2 *N* is 412. All models are fitted with full-information maximum likelihood.

*p < 0.05.

**p < 0.01.

***p < 0.001.

Given the greater proportion of women in our sample, there is a concern about the different statistical power between the two groups. To address the issue, we conducted a sensitivity analysis for H3 by randomly dropping 100 women to have an equal sample size for men and women. The results were still consistent with both H3a and H3b, and we found significant interaction effects between perceived organizational tightness and gender on job demands (γ = 0.40, *SE* = 0.16, *p* = 0.011; *simple slope* = 0.22, *SE* = 0.11, *p* = 0.047 for women vs. *simple slope* = −0.19, *SE* = 0.12, *p* = 0.11 for men) and job resources (γ = −0.42, *SE* = 0.21, *p* = 0.045; *simple slope* = 0.10, *SE* = 0.14, *p* = 0.48 for women vs. *simple slope* = 0.52, *SE* = 0.15, *p* = 0.0007 for men).

The relation between job demands and exhaustion was significant (γ = 0.29, *SE* = 0.04, *p* < 0.0001), while the relation between perceived organizational tightness and exhaustion was not (γ = 0.02, *SE* = 0.06, *p* = 0.70). Supporting H4a, the indirect effect of perceived organizational tightness on exhaustion via job demands was significant for women [*estimate* = 0.09, 95% CI (0.04, 0.15)] but not for men [*estimate* = −0.05, 95% CI (−0.13, 0.01)]. Job resources were associated with disengagement significantly (γ = −0.08, *SE* = 0.03, *p* = 0.016). The relation between perceived organizational tightness and disengagement was not significant (γ = 0.04, *SE* = 0.06, *p* = 0.57). Supporting H4b, the indirect effect of perceived organizational tightness on disengagement via job resources was significant for men [*estimate* = −0.04, 95% CI (−0.10, −0.01)] but not for women [*estimate* = −0.01, 95% CI (−0.03, 0.01)]. Overall, the results showed that women who perceived their organizational culture as tight reported higher job demands and greater exhaustion, while men in the same environment reported increased job resources and reduced disengagement.

### Discussion

Perceived organizational tightness was positively associated with both job demands and resources, which were further associated with greater exhaustion but lower disengagement, respectively. The findings supported our proposition that perceived organizational tightness serves as a double-edged sword for employee wellbeing. Most importantly, women experienced greater job demands but fewer job resources as they perceived the organizational culture to be tight (versus loose). Consequently, women experienced greater exhaustion compared to men when they perceived organizations to be tighter. By contrast, the indirect effect of perceived organizational tightness via job resources on disengagement was significant for men. This indicates that men reported lower disengagement owing to greater job resources when they perceived organizations to be tighter.

In sum, the findings from Study 1 lend support to all hypotheses. However, there are two main limitations, which we aimed to address in our second study. First, we adopted single-item measures for job demands (physical workload) and job resources (performance feedback). However, the type of job demands and resources that might be relevant to employees in tight organizational cultures likely go beyond those measured in Study 1. Therefore, it is necessary to ensure a broader coverage of job demands and resources in the next study (e.g., excessive work pressure, work-related support from colleagues). Second, the study lacks evidence of causality. While previous research has empirically demonstrated the causal effects of job demands and resources on burnout (Bakker et al., 2023), it is still necessary to establish internal validity with regard to the relation between perceived organizational tightness and job demands and job resources, as well as the moderating effect of gender. To address these limitations and complement our findings in Study 1, we conducted another study in an experimental setting to replicate the results for H1 to H3.

## Study 2

In Study 2, we used an experimental design to test the causal pathways from organizational cultural tightness to job demands and job resources. The study was pre-registered at AsPredicted #176051 (the pre-registration is available on the OSF).

### Participants and procedure

An *a priori* power analysis using G*Power 3.1 (Faul et al., 2007, 2009) provided a minimum of 787 participants as a sample size based on the following parameters: *F* tests; analysis of variance (ANOVA) (fixed effects, special, main effects and interactions); *f* = 0.10 (small effect size); α = 0.05, Power (1 −β) = 0.80; numerator *df* = 1; number of groups = 4. Accounting for possible attrition and invalid responses, we recruited 800 working adults in the U.S. via Prolific. Participants were compensated with 1.2 USD for successfully completing the study.

As per the pre-registration, we excluded 157 respondents who failed the manipulation check item for gender (*N* = 15), provided all identical ratings to the items for job demands and job resources (i.e., straight lining; *N* = 46), and did not work part-time or full-time in a company (*N* = 96; e.g., students, self-employed, home-makers). Our final sample thus consisted of 643 participants (*M*_age_ = 41.75, *SD*_age_ = 11.81; 50.54% women; 68.58% White). Participants worked in various sectors such as professional, scientific, or technical services (42.77%), manufacturing (14.93%), and educational services (11.51%).

We employed a between-subject 2 (organizational tightness: tight vs. loose) × 2 (gender: man vs. woman) factorial design. Following the consent to participate in the study, participants were randomly assigned to read a vignette about a working adult who was either a man (Adam) or woman (Anna) and had been working in a hypothetical company that either had a tight or loose corporate culture. The organizational tightness manipulation was adapted from Ma et al. (2023). The details of the manipulation are presented in the Supplementary material (pp. 5–6).

Afterward, participants were asked to indicate the gender of the employee in the vignette (man or woman) as a manipulation check for gender. As a manipulation check for organizational tightness, participants were then prompted to rate the extent to which it is important for the company to have many norms and rules for employees (−3 = *Unimportant* to + 3 = *Important*). Thereafter, participants answered three items for job demands and job resources, respectively. The items were presented in a random order within the scales for each participant. The measure for job demands included: “Adam/Anna is required to work hard,” “Adam/Anna faces conflicting demands,” and “Adam’s/Anna’s job entails an excessive workload” (adapted from Karasek, 1979; α = 0.72). Job resources were rated with the following items: “Adam/Anna receives regular feedback on his/her performance from his/her colleagues,” “Adam’s/Anna’s colleagues communicate with him/her about his/her duties and responsibilities at work,” and “Adam/Anna receives information from his/her colleagues about how to perform his/her work tasks” (adapted from Morgeson and Humphrey, 2006 and Sawyer, 1992; α = 0.87). All items were rated on a 5-point scale (1 = *Strongly disagree* to 5 = *Strongly agree*).

### Results

As mentioned above, 15 participants (8 participants in the man condition, 7 participants in the woman condition) failed to correctly identify the gender of the employee in the scenario. A chi-square test for independence with a 2 (manipulated gender) × 2 (gender indicated by participants) matrix demonstrated that participants chose correct gender significantly better than chance alone, χ^2^ (1) = 737.28, *p* < 0.0001. Regarding manipulation check for organizational tightness, participants in the tight (vs. loose) condition reported that the company values having many norms and rules to a greater extent, demonstrating that our manipulation worked successfully [*M* = 2.23, *SD* = 1.10, 95% CI (2.11, 2.35) in the tight condition vs. *M* = −1.11, *SD* = 2.12, 95% CI (−1.34, −0.88) in the loose condition], *t*(641) = 25.16, *p* < 0.0001, Cohen’s *d* = 1.98.

Consistent with H1, participants in the tight organization condition perceived greater job demands [*M* = 3.83, *SD* = 0.68, 95% CI (3.76, 3.91)] than did those in the loose organization condition [*M* = 3.33, *SD* = 0.93, 95% CI (3.23, 3.43)], *t*(641) = 7.83, *p* < 0.0001, Cohen’s *d* = 0.62. Also, supporting H2, the results showed that participants in the tight organization condition perceived greater job resources [*M* = 3.85, *SD* = 0.85, 95% CI (3.76, 3.94)] than did those in the loose organization condition [*M* = 3.05, *SD* = 1.11, 95% CI (2.92, 3.17)], *t*(641) = 10.32, *p* < 0.0001, Cohen’s *d* = 0.81.

**FIGURE 4:**
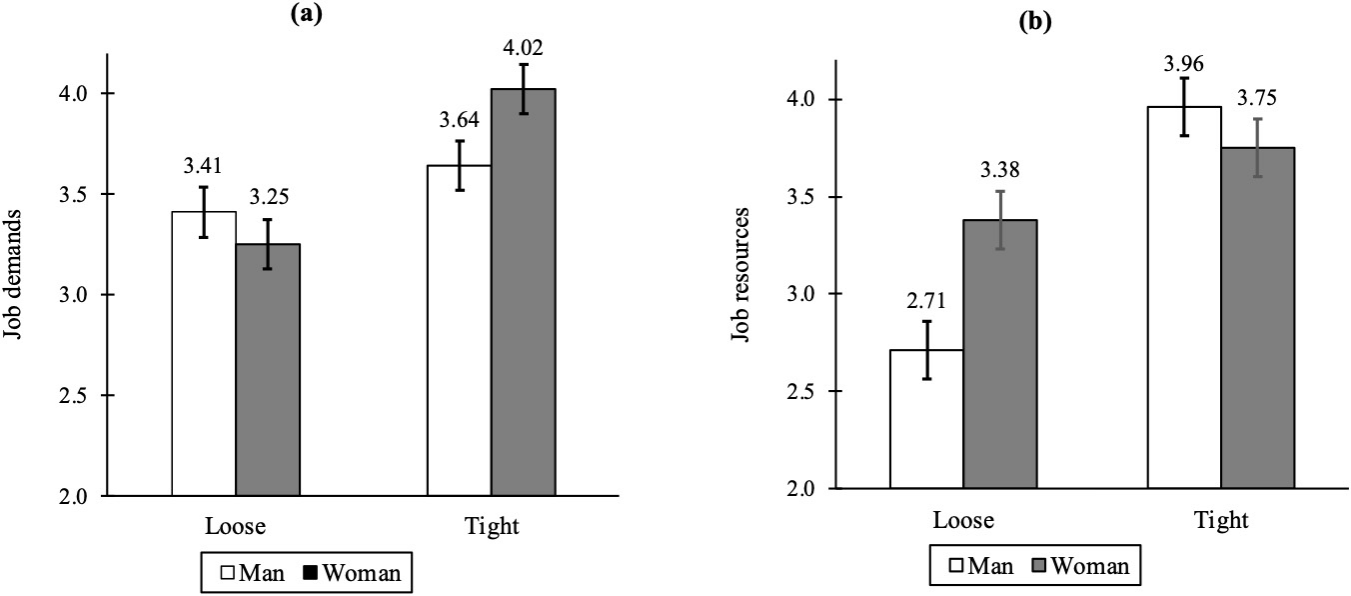
Interaction effects on **(a)** job demands and **(b)** job resources in study 2. The error bars represent 95% confidence intervals around the means.

To test the interaction between organizational tightness and gender, we conducted 2 × 2 Type-III tests of ANOVA for job demands (H3a) and job resources (H3b) to account for the unbalanced sample sizes across the conditions. Organizational tightness manipulation had a significant main effect on job demands, *F*(1, 639) = 6.55, *p* = 0.011, $\eta_{p}^{2}$ = 0.010, whereas gender did not, *F*(1, 639) = 2.91, *p* = 0.089, $\eta_{p}^{2}$ = 0.005. The interaction between the organizational tightness and gender manipulations was significant, *F*(1, 639) = 18.19, *p* < 0.0001, $\eta_{p}^{2}$ = 0.028. As shown in Figure 4a, the difference in job demands between the loose and tight organization conditions was greater within the woman condition [*M* = 3.25, *SE* = 0.06, 95% CI (3.13, 3.38) in the loose organization condition vs. *M* = 4.02, *SE* = 0.06, 95% CI (3.90, 4.14) in the tight organization condition; *contrast* = 0.77, *SE* = 0.09, *t*(639) = 8.63, *p* < 0.0001] than the man condition [*M* = 3.41, *SE* = 0.06, 95% CI (3.28, 3.53) in the loose organization condition vs. *M* = 3.64, *SE* = 0.06, 95% CI (3.51, 3.76) in the tight organization condition; *contrast* = 0.23, *SE* = 0.09, *t*(639) = 2.56, *p* = 0.011]. While the difference in job demands between man and woman within the loose organization condition did not differ significantly [*contrast* = 0.15, *SE* = 0.09, *t*(639) = 1.71, *p* = 0.089], job demands were significantly higher for woman than for man within the tight organization condition [*contrast* = 0.39, *SE* = 0.09, *t*(639) = 4.34, *p* < 0.0001].

Concerning job resources, both organizational tightness [*F*(1, 639) = 135.04, *p* < 0.0001, $\eta_{p}^{2}$ = 0.174] and gender manipulations [*F*(1, 639) = 39.45, *p* < 0.0001, $\eta_{p}^{2}$ = 0.058] had significant main effects. The interaction between the organizational tightness and gender manipulations was also significant, *F*(1, 639) = 34.09, *p* < 0.0001, $\eta_{p}^{2}$ = 0.051. As in Figure 4b, job resources were significantly higher for man in the tight organization condition [*M* = 3.96, *SE* = 0.08, 95% CI (3.81, 4.11)] compared to the loose organization condition [*M* = 2.71, *SE* = 0.08, 95% CI (2.56, 2.86)], *contrast* = 1.25, *SE* = 0.11, *t*(639) = 11.62, *p* < 0.0001. While job resources were also higher for woman in the tight organization condition [*M* = 3.75, *SE* = 0.07, 95% CI (3.60, 3.89)] than in the loose organization condition [*M* = 3.38, *SE* = 0.08, 95% CI (3.23, 3.53)], the difference between the conditions was smaller [*contrast* = 0.36, *SE* = 0.11, *t*(639) = 3.42, *p* = 0.0007]. In the loose organization condition, job resources were higher for woman (vs. man) [*contrast* = 0.67, SE = 0.11, *t*(639) = 6.28, *p* < 0.0001], while man (vs. woman) was perceived to experience more job resources within the tight organization condition [*contrast* = 0.21, *SE* = 0.11, *t*(639) = 1.96, *p* = 0.050]. In toto, the results support both H3a and H3b.

### Discussion

In Study 2, we addressed a concern for the single-item measures in Study 1 by using more general measures for job demands and job resources. Our findings from the two studies were consistent when we captured different aspects of job demands and job resources. While the findings in the previous study are limited to physical workload and performance feedback, we found more robust evidence in Study 2 showing that organizational tightness positively affects job demands and job resources and that its impacts differed for men and women. More importantly, Study 2 addressed the issue of internal validity in Study 1. Using an experimental design, we were able to establish causal inference with regard to the main effects of perceived organizational tightness and the moderating effects of gender on job demands and job resources, which complements our findings in Study 1.

## General discussion

Drawing on the literature of cultural tightness and JD-R model (Demerouti et al., 2001; Di Santo et al., 2021; Harrington and Gelfand, 2014), we theorized that perceived organizational tightness influences burnout through two mechanisms: job demands and job resources. Further, we proposed that gender moderates these relationships, such that perceived organizational tightness increases job demands for women and job resources for men, resulting in more burnout for women than for men. The results from the daily diary study (Study 1) and pre-registered experiment (Study 2) supported our theoretical model. Study 1 demonstrated that female (vs. male) employees experienced greater burnout via the dual pathways of job demands and resources when they perceived organizational culture to be tighter. Study 2 further strengthened this causal claim by showing that perceived organizational tightness had differential impacts on job demands and resources between male and female employees. Integrating the findings from the two studies, we highlight the nuanced implications of perceived organizational tightness for men and women and contribute important insights to the ongoing debate about whether tight cultures benefit or impair wellbeing. Below, we discuss the theoretical and practical implications of our results.

### Theoretical implications

Our research makes several theoretical contributions at the intersection of organizational tightness, burnout, and gender research. First, we show that organizational tightness, often viewed as a force that unifies employee behaviors, can actually lead to divergent reactions regarding employee wellbeing. The JD-R perspective sheds light on the dual pathways through which organizational tightness can both increase and decrease burnout. Prior research has tended to view organizational tightness as either beneficial or detrimental (Di Santo et al., 2021; Huang and Ren, 2017; Shi et al., 2023), without considering its simultaneous, functional and dysfunctional implications for employee outcomes. Indeed, Demerouti and Bakker (2023) recently noted that governmental regulations during COVID-19–which may be comparable to tight organizational norms–functioned as organizational demands (e.g., implementation of health and safety measures) and as resources (financial support). Departing from this either/or discussion, our findings show that perceived organizational tightness can benefit and harm employee wellbeing concurrently, rather than having unilaterally positive or negative effects in one situation over another.

Second, our study found that the impact of perceived organizational tightness on employee wellbeing varies by gender. For men, perceived organizational tightness increased job resources, which reduced their disengagement; for women, perceived organizational tightness increased job demands, which amplified their exhaustion. Importantly, these gender-based effects persisted even after controlling for the effects of some industries known for high job demands, such as healthcare and education. Our findings supported the view that tight organizational cultures intensify gender disparities, rather than hold standards and expectations constant. Despite increasing women’s leadership across various organizations over time, the inclination toward agency in the corporate world remains deeply entrenched in the society, as partially represented by the *think manager-think male* effect (Heilman et al., 2024; Schein, 1973). In line with prior findings about the challenges faced by women in tight societies (Egan et al., 2022; Toh and Leonardelli, 2012; Qin et al., 2023), we reveal that tight organizational cultures also have important implications in terms of more detrimental wellbeing for women than for men. Such divergent outcomes present a caveat that the notion of “men as cultural ideals” (Cuddy et al., 2015) can be more strongly reinforced in tight organizations.

Third, beyond the literatures of cultural tightness and burnout, we also contribute to the JD-R model by identifying perceived organizational tightness as a proximal driver of job demands and resources. Job demands and resources are often conceptualized in terms of the working conditions (Bakker et al., 2014; Crawford et al., 2010), but as in our study, they are commonly measured via self-reports (Häusser et al., 2010) in the form of subjective appraisals of job demands and resources. As the influence of subjective appraisals of the work conditions on employees’ affect and behavior is well-established (e.g., stress, task performance; Lazarus and Folkman, 1984; Lepine et al., 2016), there has also been a call for more research on what predicts these subjective appraisals (Crawford et al., 2010; Foulk and Lanaj, 2022; Lepine et al., 2005). Recent advancement of the JD-R model (Bakker et al., 2023; Demerouti and Bakker, 2023) elucidates that employees can influence their job demands and resources through proactive behaviors or self-undermining. However, research remains limited on how their assessments of organizational climate and culture may translate into job demands and resources, with psychosocial safety climate, which concerns employees’ perceptions of safety management in organizations, being one of the few notable exceptions (e.g., Dollard and Bakker, 2010; Idris et al., 2014; Loh et al., 2025). Our study thus takes an important step toward extending the JD-R model by demonstrating how perception of tight organizational cultures affects one’s appraisals of the job demands and resources.

### Practical implications

As of 2021, nearly 50% of employees experience burnout globally (McKinsey, 2021). According to Google Trends (Google, 2024), the search frequency of the term “burnout” has almost doubled globally compared to 10 years ago (Supplementary Figure 1). As burnout poses a serious threat to organizations by impairing one’s work performance and mental health (Halbesleben and Bowler, 2007; Toker and Biron, 2012), the need to understand its antecedents and remedies has continued to surge in management practices.

In this regard, our work provides several important practical implications to management of organizational cultures. Previous research offers the recommendations that fostering certain types of climate/culture, workplace politics, as well as leadership (e.g., psychosocial safety climate, transformational leadership, work performance norms) can improve employees’ work conditions, which subsequently affect various indicators of employee wellbeing such as exhaustion, engagement, depression, job stress, and irritation (Afsharian et al., 2018; Hentrich et al., 2017; Idris et al., 2014; Rennesund and Saksvik, 2010). Our study offers a caveat to these recommendations. Though enforcing these norms may translate to job resources, *tightening* organizations (i.e., designing strict attendance and monitoring policies and imposing punishments for deviance) likely conduces to job demands. However, this caveat does not equate with the notion that organizations should *loosen* their cultures entirely by eliminating these organizational norms, considering the benefits of tight organizational cultures on job resources. Rather, organizations could take an ambidextrous approach where managers maintain an optimal balance of tightness and looseness (e.g., Parker, 2014). For example, managers can stop relying on punishments and instead adopt a motivational strategy of positive and negative reinforcements for following organizational norms (e.g., amplifying job resources and reducing job demands for taking actions congruent with the norms). Another potential strategy may be concerned with behavioral nudges, which facilitates employees’ norm compliance without severe sanctions for breaking the norms (Wu and Paluck, 2021). Such strategies can help organizations reinforce the norms for desired attitudes and behaviors, while preventing the norms from becoming part of job demands to employees.

Furthermore, organizations need to be careful about the backlash from women when enforcing tight organizational cultures. Specifically, organizations can take a step toward optimizing job demands and resources for women through redesigning their work environments (e.g., developing a system for frequent informal feedback, providing social support from supervisors and coworkers) (Bakker et al., 2014). Also, we suggest job crafting as an individual-level, bottom-up coping intervention for women who are embedded in tight organizational cultures. Referring to self-initiated, physical, and cognitive changes employees make to various aspects of their jobs (Wrzesniewski and Dutton, 2001), job crafting enables employees to proactively change their work environments and manage their job demands and resources, which consequently enhances work engagement and job satisfaction (Bakker et al., 2023; Tims et al., 2012, 2013). Based on our findings, we recommend that tight organizations offer job crafting training tailored specifically to the needs and skill requirements for women so they can manage their job demands and resources more effectively.

### Limitations and future directions

Our study entails several limitations that may be addressed in future research. The first issue is common method variance, which is attributed to self-report measures for our focal variables. Job demands and resources as well as burnout are typically considered subjective perceptions and measured via self-reports (e.g., Crawford et al., 2010; Demerouti et al., 2015). Also, supervisors and coworkers are unlikely to observe a focal employee throughout the entire day (Foulk and Lanaj, 2022). As such, we opted for self-reports over third-party ratings in our research with the daily design. Additionally, our data collection in Study 1 occurred at multiple occasions across days, and this is advantageous over a traditional survey method in terms of addressing common method variance (Beal, 2015). Nevertheless, replicating our findings with objective measurement of work characteristics or expert ratings on job demands and resources (Häusser et al., 2010; Rau et al., 2010) may be an avenue for future research.

Another limitation concerns the measurement of job demands and resources in Study 1. We used a single-item measure for physical workload (job demands) and for feedback (job resources) in Study 1 to minimize participants’ burden during the mid-day surveys and thereby improve their response rates. Although we chose the items that were shown to be most representative of job demands and resources, respectively, in the original JD-R framework (See Demerouti et al., 2001), we acknowledge that the reliance on these single-item measures may have compromised the validity of our findings. We addressed this limitation by adopting the multi-item scales for both variables and assessing the influence of perceived organizational tightness on various dimensions of job demands (high workload, conflicting demands) and resources (feedback, role clarity) in Study 2. Nonetheless, we did not measure other aspects (autonomy, time pressure, rewards, supportive leadership, and job security) (Demerouti et al., 2001) in our studies, raising the question of whether perceived organizational tightness would consistently increase job demands and resources across these different dimensions. We thus encourage future research to include other types of job demands and resources that were not assessed in our studies to further enhance generalizability of our findings and to develop a more nuanced understanding about how tight versus loose cultures may impact employee wellbeing.

Also, our samples in both studies were recruited from the Western (the U.S. and U.K.) populations. Given that tightness in societal norms can vary within a country in both Western and non-Western nations (Chua et al., 2019; Harrington and Gelfand, 2014), our theorization about individual-level perceptions of organizational tightness may be still considered to be relevant across national cultures. However, as our participants are in general exposed to moderate levels of tightness in everyday social situations (Gelfand et al., 2011), a lack of sample diversity may have potentially confounded with organizational tightness they experienced at work in our studies. Future studies should thus investigate the relevance and generalizability of our research and test a cross-level interaction between perceived organizational tightness and nation-level cultural tightness in non-Western contexts and/or the societies with different levels of cultural tightness.

While our work extends organizational tightness to individual level, employees may not simply differ on perceived organizational tightness, but they may also desire different levels of organizational tightness. Recent work has demonstrated that those who *desire*—rather than perceive—organizational tightness tend to exert greater self-control and exhibit more negative emotional reactions toward organizational deviance (Mula et al., 2021; Mula and Pierro, 2022). Collectively, these findings and our research raise the possibility that employees may experience either correspondence or discrepancy between perceived and desired levels of organizational tightness. As suggested by the person-environment fit literature (e.g., Edwards, 2008; Kristof-Brown et al., 2023), organizations are not always able to satisfy individual needs and values. Future research should thus examine individual perceptions of and desire for organizational tightness simultaneously to better understand their joint impacts on employee attitudes and behaviors.

Lastly, we encourage future research to go beyond employee wellbeing as an outcome variable of tight organizational cultures. For example, as mentioned above, tight cultures are often considered to be detrimental to creativity and innovation (e.g., Chua et al., 2015; Farmer et al., 2024; Harrington and Gelfand, 2014; Shi et al., 2023). However, we suggest a more nuanced view toward the negative influence of tight cultures on creativity. In light of our findings, men might find more opportunities for creative ideas in tight organizational cultures via greater access to job resources. Gedik and Ozbek (2020) demonstrated that cultural tightness curtailed employee creativity when team collectivism was low but not when it was high. The findings from our and Gedk and Ozbek’s studies offer suggestive evidence that organizational tightness might hamper employee creativity when individuals may need to possess conflicting beliefs and values. Future research can delve into how tight organizational cultures affect employee creativity through the mechanisms of job demands and resources and whether employees’ personal fit with tight organizational cultures would influence their creative endeavors.

## Conclusion

We demonstrate that perceptions of tight organizational cultures simultaneously promote and undermine employee burnout through the competing mechanisms of job demands and resources. Tight organizational cultures impose higher demands on women, increasing exhaustion, while men benefit from enhanced resources that reduce disengagement. Although tight organizational cultures often offer structural benefits and simplify the management of employees, their influence is rarely uniformly positive. Organizations considering tighter structures should thus implement interventions to mitigate their downsides. For example, informal feedback and social support can balance demands and resources, behavioral nudges can encourage compliance without penalties, and job crafting training can help employees proactively manage demands and boost engagement. Drawing on the dual impacts of organizational tightness, it is crucial for managers to carefully develop such interventions to fully appreciate the value of tight organizational cultures.

## Data Availability

The datasets presented in this study can be found in online repositories. The names of the repository/repositories and accession number(s) can be found in the article/Supplementary material.

## Footnotes

1 Throughout the manuscript, we refer to gender as a social construct, encompassing “the attitudes, feelings, and behaviors that a given culture associates with a person’s biological sex” (American Psychological Association, 2022). Accordingly, we use man/woman and male/female to refer to a person’s identified gender rather than their biological sex.

2 As an attention check, participants were given a paragraph, which included an instruction that they needed to type a specific word in the question that followed: “Based on the text above, what food have you been asked to enter?” Those who failed to enter the word were excluded from analyses.

3 We had a lower response rate on Tuesday compared to other days, as we were initially using an incorrect survey link. Upon noticing the glitch, we promptly addressed it by sending out a new survey link to the participants.

4 Results did not change substantially when we tested the hypotheses only with full-time employees (see Supplementary Tables 1, 2).
