# Supplementary material for: Tight cultures as a double-edged sword for burnout: the job demands-resources perspective on perceived organizational tightness and the moderating effect of gender
Source: Front Psychol. 2026 Jan 7;16:1642855. doi: 10.3389/fpsyg.2025.1642855 (PMC12819331; doi:10.3389/fpsyg.2025.1642855)
Supplement: Supplementary file 1 [file Data_Sheet_1.pdf]

Supplemental Materials

For

Tight Cultures as a Double-Edged Sword for Burnout: The Job Demands-Resources Perspective on Perceived Organizational Tightness and the Moderating Effect of Gender

Table of Contents

Measures and Stimuli 3

Study 1 3

Study 2 5

Additional Analyses 8

Table S1 8

Table S2 9

Figure S1 10

References 11

Measures and Stimuli

Study 1

Initial Survey

- Perceived organizational tightness (adapted from Gelfand et al., 2011; 6-point scale; *Strongly disagree* to *Strongly agree*)
    1. There are many strong norms that people are supposed to abide by in my organisation.
    2. In my organisation, there are clear norms for how people should act in most situations.
    3. People agree upon the norms for what behaviours are considered appropriate versus inappropriate in my organisation.
    4. In most situations, norms in my organisation give people a great deal of freedom in deciding how they want to behave. (Reverse-scored)
    5. In my organisation, if someone acts in a way that deviates from the norms, others will strongly disapprove.
    6. People in my organization almost always comply with norms at work.

Morning Surveys

- Positive affect (adapted from MacKinnon et al., 1999; 5-point scale; *Not at all* to *Very much*)
    1. Right now, I feel inspired.
    2. Right now, I feel alert.
    3. Right now, I feel excited.
    4. Right now, I feel enthusiastic.
    5. Right now, I feel determined.

- Negative affect (MacKinnon et al., 1999; 5-point scale; *Not at all* to *Very much*)
    1. Right now, I feel afraid.
    2. Right now, I feel upset.
    3. Right now, I feel nervous.
    4. Right now, I feel scared.
    5. Right now, I feel distressed.

Afternoon Surveys

- Job demands and job resources (adapted from Schönfelder, 1992; Hackman & Oldham, 1975; Demerouti et al., 2001; 5-point scale; *Not at all* to *Very much*)
    1. Since the last survey, I feel it is physically taxing for me to get used to my working time.
    2. Since the last survey, I feel I get enough feedback about the quality of my performance.

Evening Surveys

- Burnout (adapted from Demerouti & Bakker, 2010; 5-point scale; *Not at all* to *Very much*)
    1. Right now, I feel emotionally drained.

2. Right now, I feel…worn out and weary,
3. Right now, I think less at work and do my job almost mechanically.
4. Right now, I feel sickened by my work tasks.

Study 2

Experimental Scenario Stimuli (adapted from Ma et al., 2023)

- Man in tight organization condition

Adam has been working at John Smith Group for five years. He is known as a **proactive** and **ambitious** individual to his colleagues. When a problem arises at work, Adam takes an **assertive** approach by **clearly sharing his opinions** with his colleagues. Other employees trust him and often reach out to him if they encounter issues at work.

At John Smith Group, Adam experiences **many social rules and norms**. For example, John Smith Group has a strict set of employee etiquette. Adam needs to always follow dress codes.

When employees work in teams, the culture requires **following established rules and procedures**. All team members need to **follow** the existing rules. This means that everyone has to be meticulous and methodical. Employees who **closely follow the rules and procedures** are **rewarded** as well.

As Adam is **ambitious and competitive**, he is motivated to advance his career in the company. Given the **presence of strict rules and norms**, Adam uses them as guidance to avoid overstepping while figuring out the best course of action at work. Appreciating the structure this culture brings, Adam is able to map out his career plan to achieve **personal success** at John Smith Group.

- Man in loose organization condition

Adam has been working at John Smith Group for five years. He is known as a **proactive** and **ambitious** individual to his colleagues. When a problem arises at work, Adam takes an **assertive** approach by **clearly sharing his opinions** with his colleagues. Other employees trust him and often reach out to him if they encounter issues at work.

At John Smith Group, Adam is expected to **smash all social rules and norms**. For example, John Smith Group has almost no social norms concerning employee etiquette. Adam wears whatever he wants at work.

When employees work in teams, the culture requires **innovation and out-of-the-box thinking**. All team members need to **challenge** the existing rules. This means that everyone has to be **creative and imaginative**. Employees **will not be punished** even when they **get off the track** during the teamwork.

As Adam is **ambitious and competitive**, he is motivated to advance his career in the company. Given the **lack of strong rules and norms**, Adam is left to navigate his career trajectory without clear markers of success. While Adam appreciates the autonomy this culture brings, he often finds himself questioning how to achieve **personal success** at John Smith Group.

- Woman in tight organization condition

Anna has been working at John Smith Group for five years. She is known as a **caring** and **empathetic** individual to her colleagues. When a problem arises at work, Anna takes a **considerate** approach by **meticulously addressing the concerns** of her colleagues. Other employees trust her and often reach out to her if they encounter issues at work.

At John Smith Group, Anna experiences **many social rules and norms**. For example, John Smith Group has a strict set of employee etiquette. Anna needs to always follow dress codes.

When employees work in teams, the culture requires **following established rules and procedures**. All team members need to **follow** the existing rules. This means that everyone has to be meticulous and methodical. Employees who **closely follow the rules and procedures** are **rewarded** as well.

As Anna is **empathetic and collaborative**, she feels responsible for advancing her team's success in the company. Given the **presence of strict rules and norms**, Anna endures exacerbated pressure to avoid overstepping while painstakingly dealing with her team's various needs and concerns. While Anna appreciates the structure this culture brings, she often finds herself pushing to find new ways to ensure **collective success** at John Smith Group.

- Woman in loose organization condition

Anna has been working at John Smith Group for five years. She is known as a **caring** and **empathetic** individual to her colleagues. When a problem arises at work, Anna takes a **considerate** approach by **actively listening to** her colleagues. Other employees trust her and often reach out to her if they encounter issues at work.

At John Smith Group, Anna is expected to **smash all social rules and norms**. For example, John Smith Group has almost no social norms concerning employee etiquette. Anna wears whatever she wants at work.

When employees work in teams, the culture requires **innovation and out-of-the-box thinking**. All team members need to **challenge** the existing rules. This means that everyone has to be **creative and imaginative**. Employees **will not be punished** even when they **get off the track** during the teamwork.

As Anna is **empathetic and collaborative**, she feels responsible for advancing her team's success in the company. Given the **lack of strong rules and norms**, Anna leverages the freedom to support and help her colleagues. Appreciating the autonomy this culture brings, Anna is able to find many ways to ensure **collective success** at John Smith Group.

Manipulation Check Items

- What is the gender of the employee you just read about? (*Male* or *Female*)
- To what degree it is important for John Smith Group to have many norms and rules for employees? (-3 = *Unimportant* to 3 = *Important*)

Measures for Job Demands and Job Resources

- Job demands (adapted from Karasek, 1979; 5-point scale; *Strongly disagree* to *Strongly agree*)
    1. In John Smith Group, Adam/Anna is required to work hard.
    2. In John Smith Group, Adam/Anna faces conflicting demands.
    3. In John Smith Group, Adam's/Anna's job entails an excessive workload.

- Job resources (adapted from Morgeson & Humphrey, 2006; Sawyer, 1992; 5-point scale; *Strongly disagree* to *Strongly agree*)
    1. In John Smith Group, Adam/Anna receives regular feedback on her performance from her colleagues.
    2. In John Smith Group, Adam's/Anna's colleagues communicate with her about her duties and responsibilities at work.
    3. In John Smith Group, Adam/Anna receives information from her colleagues about how to perform her work tasks.

Additional Analyses

Table S1

Supplementary Analyses for Full-Time Employees in Study 1

	Job demands		Job resources		Job demands		Job resources		Exhaustion		Disengagement	
Variables	*γ*	*SE*	*γ*	*SE*	*γ*	*SE*	*γ*	*SE*	*γ*	*SE*	*γ*	*SE*
Intercept	1.84***	0.27	2.75***	0.34	1.85***	0.26	2.75***	0.34	1.80***	0.25	2.82***	0.25
Within-person level variables												
Job demands	-	-	-	-	-	-	-	-	0.17***	0.04	-	-
Job resources	-	-	-	-	-	-	-	-	-	-	-0.01	0.02
Day	-0.02	0.01	-0.02†	0.01	-0.02	0.01	-0.02†	0.01	-0.03*	0.02	-0.01	0.01
Positive affect	-0.12***	0.03	0.03	0.03	-0.12***	0.03	0.03	0.03	-0.03	0.04	-0.03	0.02
Negative affect	0.03	0.04	-0.06	0.04	0.03	0.04	-0.06	0.04	0.18***	0.05	0.12***	0.03
Between-person level variables												
Job demands	-	-	-	-	-	-	-	-	0.28***	0.04	-	-
Job resources	-	-	-	-	-	-	-	-	-	-	-0.08*	0.03
Perceived organizational tightness	0.13†	0.07	0.27**	0.09	0.05	0.07	0.33***	0.10	0.03	0.06	0.05	0.06
Gender	0.03	0.09	0.17	0.11	0.04	0.09	0.17	0.11	0.13†	0.08	-0.07	0.08
Perceived organizational tightness x Gender	-	-	-	-	0.50***	0.15	-0.37†	0.19	-	-	-	-
Age	-0.00	0.01	-0.01	0.01	-0.00	0.00	-0.01	0.01	0.00	0.00	-0.00	0.00
Race	-0.00	0.11	0.14	0.14	-0.03	0.11	0.16	0.14	0.02	0.10	0.13	0.10
Tenure	-0.01	0.01	-0.00	0.01	-0.01	0.01	-0.00	0.01	-0.01	0.01	0.00	0.01
Healthcare industry	0.37**	0.14	-0.06	0.18	0.30*	0.14	-0.00	0.18	0.16	0.13	0.09	0.13
Education and training industry	0.15	0.12	-0.06	0.15	0.11	0.12	-0.03	0.15	0.05	0.11	-0.02	0.11
Positive affect	-0.18**	0.06	0.36***	0.07	-0.18**	0.06	0.35***	0.07	-0.13*	0.05	-0.25***	0.05
Negative affect	0.59***	0.07	-0.31**	0.09	0.59***	0.07	-0.31***	0.09	0.52***	0.07	0.29***	0.07
Variance components												
Within-person variance	0.35		0.43		0.35		0.43		0.63		0.21	
Intercept variance	0.59		1.00		0.57		0.99		0.36		0.48	
Random slope variance	-		-		-		-		0.01		0.01	
Model fit												
AIC	3493.6		3911.5		3484.5		3909.7		4043.3		2887.3	
BIC	3573.3		3991.1		3569.5		3994.6		4144.3		2988.2	
Log-likelihood	-1731.8		-1940.7		-1726.2		-1938.8		-2002.7		-1424.6	

Note. Level-1 N is 1,497 and level-2 N is 401. All models are fitted with full-information maximum likelihood.

† $p < .10$. * $p < .05$. ** $p < .01$. *** $p < .001$.

Table S2

Summary of Indirect Effects for Full-Time Employees in Study 1

		Men		Women	
Outcome	Mediator	Estimate	95% CI	Estimate	95% CI
Exhaustion	Within-level job demands	-0.03	-0.08, 0.01	**0.05**	0.02, 0.09
	Between-level job demands	-0.06	-0.13, 0.01	**0.08**	0.03, 0.14
Disengagement	Within-level job resources	-0.01	-0.03, 0.02	-0.002	-0.01, 0.01
	Between-level job resources	**-0.04**	-0.09, -0.01	-0.01	-0.03, 0.01

Note. Level-1 N is 1,497 and level-2 N is 401. Confidence intervals were calculated with the quasi-Bayesian Monte Carlo method.

Figure S1

Google Trends Data

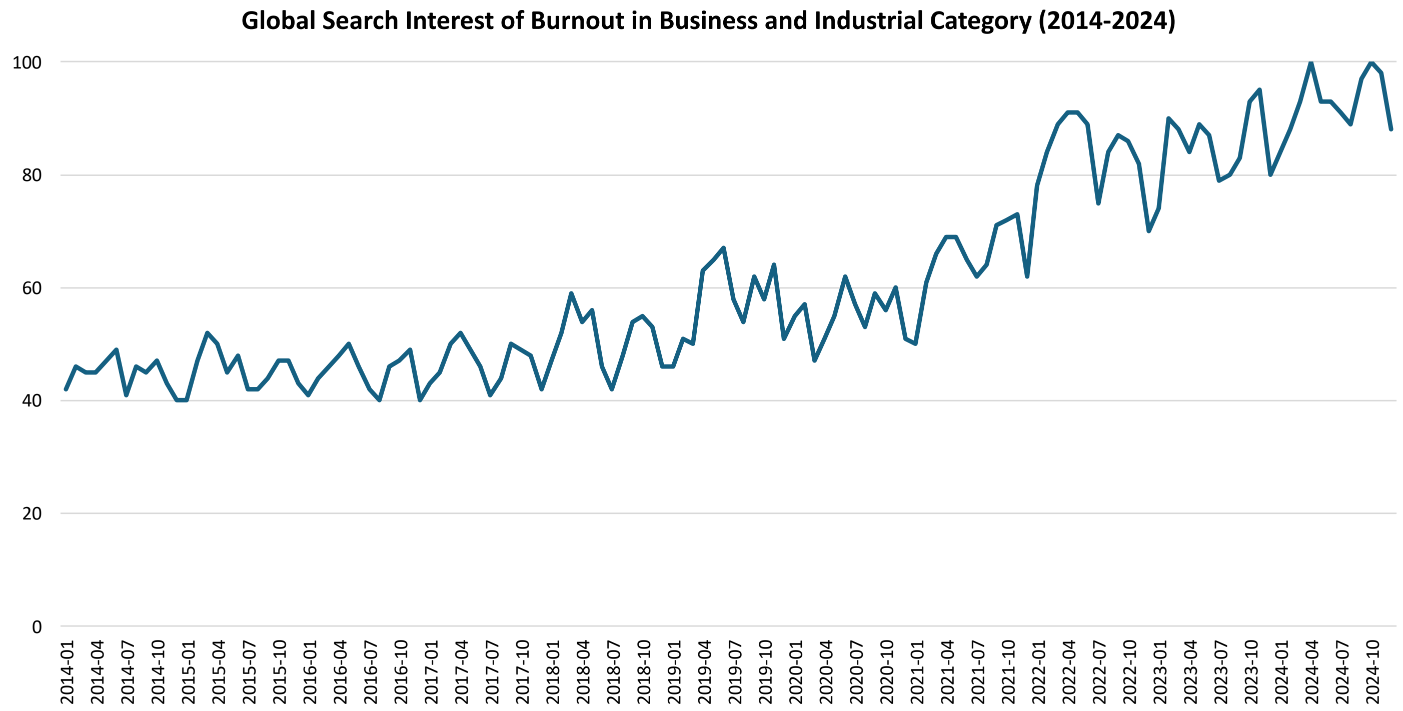

Note. Google Trends (Google, 2024) is a tool offered by Google (https://trends.google.com/trends/) that analyzes search queries submitted in Google Search. Google Trends does not provide absolute values for search queries, but it compares relative search volumes of a term over time to show trends in public interest and popularity of the term across regions and categories. Search results are normalized on a scale of 0-100, and 100 represents the highest search interest for the time and location(s) selected, allowing for a comparison of the search frequency of the term across different time points. The graph above thus shows relative popularity of the term "burnout" for the past 10 years (time range: 01/01/2014-12/31/2024) in the Business and Industrial category worldwide. The original data exported from Google Trends to construct the graph is available at OSF: https://osf.io/8wyrc/?view_only=76f80c4401954531a793f73f2e4416fd.

References

Demerouti, E., B. Bakker, A., Nachreiner, F., & Schaufeli, W. (2001). The job demands–resources model of burnout. *Journal of Applied Psychology, 86*(3), 499-512. https://doi.org/10.1037/0021-9010.86.3.499

Demerouti, E., Mostert, K., & Bakker, A. B. (2010). Burnout and work engagement: A thorough investigation of the independency of both constructs. *Journal of Occupational Health Psychology*, *15*(3), 209–222. https://doi.org/10.1037/a0019408

Gelfand, M., Raver, J., Nishii, L., Leslie, L., Lun, J., Lim, B., . . . Yamaguchi, S. (2011). Differences between tight and loose cultures: A 33-nation study. *Science, 332*(6033), 1100-1104. DOI: 10.1126/science.1197754

Google. (2024). *Google trends*. https://www.google.com/trends

Hackman, J. R., & Oldham, G. R. (1975). Development of the job diagnostic survey. *Journal of Applied Psychology, 60*(2), 159-170. https://doi.org/10.1037/h0076546

Karasek, R. A. (1979). Job demands, job decision latitude, and mental strain: Implications for job redesign. *Administrative Science Quarterly, 24*(2), 285-308. https://doi.org/10.2307/2392498

Ma, A., Savani, K., Liu, F., Tai, K., & Kay, A. C. (2023). The mutual constitution of culture and psyche: The bidirectional relationship between individuals' perceived control and cultural tightness–looseness. *Journal of Personality and Social Psychology, 124*(5), 901-916. https://dx.doi.org/10.1037/pspa0000327

Mackinnon, A., Jorm, A. F., Christensen, H., Korten, A. E., Jacomb, P. A., & Rodgers, B. (1999). A short form of the Positive and Negative Affect Schedule: Evaluation of factorial validity and invariance across demographic variables in a community sample. *Personality*

and Individual Differences, 27(3), 405-416. https://doi.org/10.1016/S0191-8869(98)00251-7

Morgeson, F. P., & Humphrey, S. E. (2006). The work design questionnaire (WDQ): Developing and validating a comprehensive measure for assessing job design and the nature of work. *Journal of Applied Psychology*, *91*(6), 1321–1339. https://doi.org/10.1037/0021-9010.91.6.1321

Sawyer, J. E. (1992). Goal and process clarity: Specification of multiple constructs of role ambiguity and a structural equation model of their antecedents and consequences. *Journal of Applied Psychology*, *77*(2), 130–142. https://doi.org/10.1037/0021-9010.77.2.130

Schönfelder, E. (1992). Entwicklung eines Verfahrens zur Bewertung von Schichtsystemen nach arbeitswissenschaftlichen Kriterien [Development of an instrument for the evaluation of shift-types according to scientific criteria]. In W. Gaul & H. G. Gemünden (Eds.), *Entscheidung-sunterstüzung für Ökonomische Probeme Bd. 3.* Lang.
